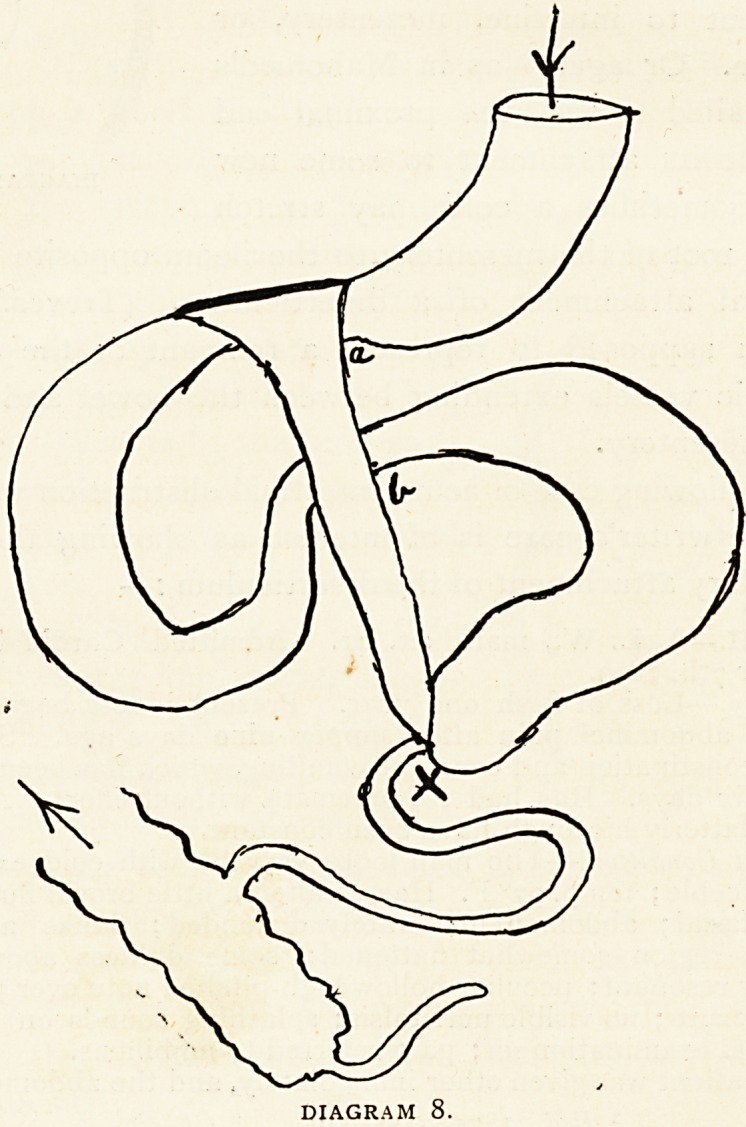# Some Surgical Aspects of Meckel's Diverticulum

**Published:** 1901-12

**Authors:** William Sheen

**Affiliations:** Surgeon to the Cardiff Infirmary.


### SOME SURGICAL ASPECTS OF MECKEL'S DIVERTICULUM.

BY

WILLIAM SHEEN, M.S., M.D. Lond., F.R.C.S.,

*Surgeon to the Cardiff Infirmary.*

THE vitelline or omphalo-mesenteric duct usually becomes obliterated in the eighth week of fœtal life, or a little earlier, and then disappears. It may persist under various aspects, the commonest of which is where its proximal end develops into a blind sac of gut some three inches long, attached to the ileum near its lower part, forming the ordinary Meckel's diverticulum, and usually giving no signs of its presence during life. Other lines of development or partial failure of involution may give rise to conditions of both clinical and pathological interest, and examples occur with sufficient frequency scattered through surgical literature to make the subject appear worthy of summary and comment. Closely associated with the duct, and producing various phenomena as a consequence of their persistence, are the omphalo-mesenteric vessels.

The following case which was under the writer's care will lead naturally to the consideration in detail of the various conditions which may be present:—

**Case I.**—E. T. L., male, æt. 1 year 9 months, admitted to the Cardiff Infirmary April 22nd, 1897.

*History.*—Swelling at navel since birth. Confinement was not attended by a doctor, and mother has been dead twelve months. Swelling has always been the same size. About half a pint of glairy fluid comes from it in twenty-four hours, staining and stiffening linen. General health always good.

*Present Condition.*—Healthy, well-nourished child. Attached to centre of navel is a bright red, bluntly lobulated, pedunculated tumour, the size of a grape, with skin reaching only to its margin. The surface resembles intestinal mucous membrane and exudes a viscid fluid of alkaline reaction. In the centre is a channel 1 inch deep. Through the parietes a cord the thickness of a cedar pencil can be felt passing backwards for about 1½ inches. Urine and fæces are normal.

After admission the fluid was collected as far as possible by a small glass vase strapped on to the child's abdomen. Amount 10—15 c.c. in twenty-four hours; on two occasions reached 22 c.c.; sometimes only 5 c.c., but then some was lost. It was a colourless viscid fluid pouring from vessel to vessel like a thin jelly, of alkaline reaction and containing a little albumin. It had no digestive action on fibrin or starch. So far as examined then, it resembled succus entericus. On July 31st the tumour was removed by scissors and the base cauterised, the procedure being quite a slight one. The child vomited continuously after the anæsthetic. On August 3rd a simple enema was given and the bowels were twice opened: on the following days the child was fretful and became thinner, the milk was peptonised but the vomiting continued; vomit undigested milk; the abdomen was distended and tender; the child worse. On August 7th, a blood-streaked motion is stated to have been passed after an enema, but it was not saved by the nurse. Nutrient enemata were given towards the end, but the child died at 5 p.m. on August 7th, one week after the operation. The cause of death was thought to be peritonitis.

August 8th. *Post Mortem.*—No peritonitis. Death found to be due to internal strangulation, and the parts involved removed for separate examination. In the specimen removed were the lower part of the small intestine, cæcum, appendix, and a small piece of ascending colon. Connected with the small intestine was a Meckel's diverticulum patent to within an inch of the umbilicus, to which it was attached by a solid cord. The skin around the umbilicus was removed by an elliptical incision.

On dissection the following points were made out:—

1. The bowel is strangulated by being herniated through a hole (A) in the mesentery of the diverticulum ilei. 2. The constricted bowel is 25 inches in length. 3. Practically all the bowel between the origin of the diverticulum and the ileo-cæcal valve is strangulated. 4. The strength of the constricting cord of mesentery is largely due to a vessel traversing it. 5. The bowel is twisted within the ring and near perforation at its proximal end. 6. The diverticulum is bulbous in shape and its lumen much narrowed where it joins the intestine.

Diagram 1 shows the condition, the strangulated loop being represented turned out of the constricting ring (A). The polypus is also

DIAGRAM I.

shown. The position of the appendix was interesting. It lay against the diverticulum, with its apex pointing towards the liver.

Microscopical examination of the polypus showed a connective tissue basis with a layer of intestinal glands—exactly like Lieberkühn's follicles. In places the intestinal glands were proliferating so as to produce a mass resembling an ordinary intestinal adenoma.

Clinically disappointing, this case is of great interest pathologically. The writer has been able to find no other record of a case of strangulation through the mesentery of a Meckel's diverticulum.[1]

**Class 1.**—Some form of remnant of the vitello-intestinal duct occurs in rather more than 2 per cent. of all bodies examined. The common form of Meckel's diverticulum (diagram 2) is a free tube of intestine ending blindly, springing from the convex border of the ileum, usually about three feet from the ileo-cæcal valve. Its lumen is that of the intestine, and its calibre is generally widest at its attached extremity. It may have a mesentery, but usually has not, deriving its nourishment from the blood-vessels in its walls. Its average length is three inches, but it has been seen as a mere bud or of the length of ten inches. It comes off from the intestine usually at right angles, sometimes obliquely. It may—very rarely—arise from the concavity of the intestine, lying within the layers of the mesentery. Internally, at its junction with the ileum, a valve may be found indicating an attempt at closure.

DIAGRAM 2.

The structure of the wall is the same as that of the small intestine. The muscular coat may be deficient at the free end, the mucous membrane being herniated under the serous coat so as to form a clubbed extremity to the diverticulum.

The diverticulum may occupy, or be strangulated in, a hernial sac; it may exhibit such morbid processes as occur in the intestine, *e.g.*, ulceration or perforation. It may produce strangulation by forming knots, especially when the extremity is clubbed. (Treves.)[2] Rolleston mentions a case in which the diverticulum became inverted into the bowel and produced an intussusception.

**Class 2.**—In a second class of cases—to which the one described above belongs—the end of the diverticulum is connected with the posterior aspect of the umbilicus by a fibrous cord which may be pervious in some part of its extent. This

[1] This case formed the subject of a paper read to the South Wales Branch of the British Medical Association.

[2] Allbutt's *System of Medicine*, vol. iii., 1897, p. 802.

may cause intestinal obstruction in various ways, the gut being thrown over it or drawn under it, or pushed through its mesentery—as in the case above detailed,—or, under certain conditions, especially much distension of the bowel, the diverticulum may drag upon it at its point of attachment, producing kinking.

DIAGRAM 3.

**Class 3.**—Similar accidents may happen when the end of the diverticulum is attached to the posterior surface of the umbilicus. (Diagram 3.) These cases may in early life have belonged to the class where a minute fistula is present, the fistula having healed spontaneously. Richardson reports a case in which an unusually large Meckel's diverticulum was attached to the ileum about three feet above the ileo-cæcal valve; its calibre was that of the ileum; it had no mesentery, but was covered with peritoneum and supplied with blood-vessels from the ileum and the abdominal wall.[1] In 1898 Heaton showed to the Midland Medical Society a patient in whom, after a short illness, a large fæcal concretion had been discharged from the umbilicus. Before and after, the patient was in perfect health. He suggested that the concretion had been formed in Meckel's diverticulum, set up ulceration and been discharged through the umbilicus.[2]

DIAGRAM 4.

**Class 4.**—We now come to the important class of cases in which the diverticulum, having been continued for a variable distance into the cord, is pervious throughout at its separation. (Diagram 4.) The entire tube may have the structure of fully-developed intestine, or a variable length nearest the umbilicus may be a channelled cord. The mucous membrane at the umbilicus, prolapsing and proliferating, often forms a little polypus, in the centre of which

[1] *Quart. M. J.*, 1894–5, iii. 267. [2] *Brit. M. J.*, 1898, i. 627.

is a minute channel through which fæcal matter extrudes. Such a case was shown by Battle to the Clinical Society in 1893. The child (a female) had an umbilical polypus with a minute opening in its centre, from which fæcal matter escaped. The bowels acted normally. The protruded portion proved to be the mucous membrane of Meckel's diverticulum.[1]

Sometimes the bowel below the attachment of the diverticulum is much contracted. Brundeau records the case of an infant which died when five days old. It was an eight months child, weighing only 2 lb. 5 oz. The diverticulum was situated 8½ inches above the cæcum. It was pervious and meconium was found in the liquor amnii. The gut was drawn forwards at a sharp angle; above the diverticulum it was much dilated, and below very small.[2]

A further stage of this condition is found when the intestine below is absolutely occluded. Its patency should always be demonstrated before attempting to close a fæcal fistula. Anderson records the case of a child with a fistula at the umbilicus through which it defæcated. After death a fistula was found in the ileum, 1½ inches from the cæcum. The intestine terminated blindly 6 inches below the ileo-cæcal valve. The case was thought to be one of incomplete hernia from non-retraction of the "navel loop," which was divided at birth. The apex of the loop was at the point of attachment of a Meckel's diverticulum which was not identified, the placental end of the cord not being preserved.[3]

If the external opening of the fistula be large the mucous membrane of the diverticulum may prolapse into it and sometimes so extensively that a spur from the ileum projects into the wound with two openings, one on each side of it. Tillmanns[4] states that in this class of case the prolapse always takes place from the proximal end of the intestine; but Golding-Bird records a case in which the ileum below the diverticulum was intussuscepted into it, the diagnosis being confirmed by an autopsy.[5]

[1] *Tr. Clin. Soc. Lond.*, 1893, xxvi. 237.

[2] *Brit. M. J.*, 1895, i. Epitome, p. 59. [3] *Tr. Path. Soc. Lond.*, 1891, xlii. 128.

[4] *A Text-Book of Surgery*, vol. iii., 1898, p. 9.

[5] *Tr. Clin. Soc. Lond.*, 1896, xxix. 32.

**Class 5.**—An interesting case, unique so far as the writer has been able to ascertain (diagram 5), was described by Heaton to the Midland Medical Society in 1895. A growth was shown removed from the navel of a boy æt. 4½ years. It was stated that when the child strained he passed water from his navel, and on examination it was found that a jet of fluid came from this situation on crying. A probe entering the fistula passed downwards and backwards for 3½ inches. The fluid was clear, light yellow, and strongly alkaline, closely resembling succus entericus. At the operation a large thick-walled, pear-shaped sac was found, passing downwards and backwards, and adherent by a band of fibrous tissue to the small intestine. The sac was excised by two operations and showed microscopically the structure of small intestine. The explanation given was that the vitello-intestinal duct had become partially obliterated, and that the remainder was distended by secretion into a thick-walled muscular sac.[1]

DIAGRAM 5.

**Class 6.**—Closure of the process at its proximal and distal extremities will give rise to cysts in or behind the umbilicus. Such conditions are very rare. (Diagram 6.) In 1887 D'Arcy Power reported a case of congenital umbilical hernia containing a foot of small intestine; the sac was transparent, being formed by the coverings of the cord with the main constituents of the funis running as a bundle along its lower border. At a short distance beyond the tumour was a small sac filled with a viscid fluid.[2] The second sac was probably a non-obliterated portion of the vitello-intestinal duct, occurring here, however, outside the body.

DIAGRAM 6.

[1] *Brit. M. J.*, 1895, ii. 1107. [2] *Tr. Path. Soc. Lond.*, 1888, xxxix. 108.

**Class 7.**—Finally, the duct may exist as a fibrous cord running from umbilicus to intestine. (Diagram 7.) F. A. Mahomed describes a case of fibrous cord which produced obstruction and death. The cord reached from the mesentery to the umbilicus, and was supposed to be a remnant of the left hypogastric artery with an abnormal origin from the ileo-colic branch of the superior mesenteric.[1] It is more probable that it was a remnant of the vitello-intestinal duct.

DIAGRAM 7.

*Secondary changes* may take place in the attachments of the duct-remnant. It may free itself from the umbilicus and remain free in the abdominal cavity, or more commonly form a secondary attachment to intestine, mesentery, or elsewhere. Or again—as in Mahomed's case detailed above—the proximal end may shift its attachment to some new point. Sometimes a cord may stretch from the root of the mesentery to the ileum opposite the point of normal attachment of a diverticulum. (Treves.)[2] This has been supposed to represent a remnant of the omphalo-mesenteric vessels extending between the bowel and the root of the mesentery.

The following case of acute intestinal obstruction which was under the writer's care is of interest as showing the results of secondary attachment of the diverticulum :—

**Case II.**—A. L. W., male, æt. 41. Admitted Cardiff Infirmary, November 7th, 1899.

*History.*—Loss of flesh one year. Present illness began with an attack of abdominal pain after supper nine days ago. Since then absolute constipation and constant vomiting, which has been fæcal for the last six days. Has had two enemata without effect. Abdominal pain and latterly hiccough have been constant.

*Present Condition.*—The man looks very ill, with cold extremities; pulse 72, feeble; temp. 97° F. Has vomited a little brown fluid matter, smelling fæcal; abdomen moderately distended; flanks and hypochondriac region somewhat flattened; some dulness above pubes; remainder resonant; peculiar hollow high-pitched note over position of sigmoid flexure; no visible peristalsis; splashing sounds on manipulation; rectal examination *nil*; pain referred to umbilicus.

The patient was given ether immediately, and the abdomen opened

[1] *Ibid.*, 1875, xxvi. 117. [2] *Op. cit.*

in the left iliac region. The colon was found empty; some distended coils of small intestine presented themselves, and the hand could feel something like a band on the right side and apparently near the pelvic brim. The closure of the wound was commenced with a view to opening in the middle line, when, somewhat suddenly, the patient—whose condition was extremely serious throughout—collapsed and died. The trachea was opened and various measures resorted to to restore animation, but without effect.

*Post Mortem.*—Twelve hours after death. Abdomen only opened through a crucial incision. No peritonitis. Small intestine distended and injected. Without disturbance the seat of obstruction was at once seen in the form of a diverticulum of the bowel passing downwards and outwards from the median line, at a point about opposite to the third lumbar vertebra, towards the pelvic brim. On examination the diverticulum, which was devoid of a mesentery, was found to be about four inches long, bulbous at its commencement, then narrowing suddenly, but patent to its extremity. It sprung from the posterior aspect of the ileum about two feet above the ileo-cæcal valve, curved forwards and inwards round the bowel from which it came, and passed downwards and inwards, to be attached by its apex to the small intestine again about five inches from the ileo-cæcal valve. (Diagram 8.)

DIAGRAM 8.

The obstruction of the ileum took place at the point of attachment of the apex of the diverticulum, which attachment was made by a few short firm adhesions. The bowel was very near perforation at this point. The gut was also pressed upon somewhat at two points above the actual seat of obstruction; viz., (1) where the diverticulum wrapped itself round the ileum at its point of origin—*a*; (2) where a loop of bowel passed under the diverticulum—*b*. It was evident that the more distended the bowel became, the more would the diverticulum pull upon and kink its point of attachment.

Reference has also been made to the possibility of an umbilical hernia persisting through traction of the omphalo-mesenteric duct. (Wm. Anderson's case.) It must be remembered that up to about the end of the third month of fœtal life umbilical hernia is a physiological condition, the greater part of the small intestine being protruded from the abdomen into the tissues of the cord forming the "navel loop," to the apex of the loop being attached the omphalo-mesenteric duct or its remnant. The following case has some bearing upon the surgical aspect of this point. Hope, at Queen Charlotte's Hospital, records that he was called to see a child just born, and found a hernial protrusion into the cord the size of a hen's egg; it was a partially reducible enterocele, the neck of which was formed by the skin and the fundus by the coverings of the cord. The umbilical vessels were spread out over the right side of the sac. The contents were found to be large and small intestine, of which five inches of the latter were adherent. The bowel was healthy and was separated without difficulty. What was believed to be the vermiform appendix was so intimately fused with the tissues of the cord, that it had to be ligatured and divided. The child recovered.[1]

If—as is probable—this cord was not the vermiform appendix, but a remnant of the omphalo-mesenteric duct, the case affords evidence of the persistence of the duct being contributory to congenital hernia. It is not probable, however, that so delicate a structure can play a very important part in the production of hernia, especially in the larger forms (exomphalos) where deficient closure of the lateral body-walls is the principal cause.

With regard to **umbilical polypus**, its presence in association with a fæcal fistula has been incidentally referred to, but the

[1] *Lancet*, 1886, ii. 773.

usual form in which a polypus exists is with its connection with the intestine cut off, as in Case I. Many such cases have been reported, but there is, as a rule, no record of any evidence of the presence of an occluded diverticulum in connection with the polypus; for their removal is simple and recovery almost invariably follows. Occasionally polypi of a different nature occur, being either in association with a persistent urachus, or resulting from proliferation of granulations after the fall of the cord or, rarely, of nævoid structure. But those arising from partial persistence of the omphalo-mesenteric duct are probably the commonest. It was some time before the true nature of these polypi was ascertained. Under the heading "Persistent Vitality of the Umbilical Cord," C. R. Williams describes a case in the *Lancet* for 1880. A baby, three weeks old, had a fleshy outgrowth, fully an inch long, projecting from the umbilicus. It was quite rigid, with a raw granulating surface, bleeding at the slightest touch and extremely sensitive. There was a small central depression at the free extremity. The nurse stated that she had to change the dressing frequently, on account of a watery oozing from the growth. It was removed by ligature round the base.[1]

Francis Villar describes a number of cases and gives references. He states that the growths were first correctly described by Kolaczek in 1875, and designated "entero-teratomes."[2] They have also been styled "adenomata of the new-born" and "prolapse of Meckel's diverticulum."

W. S. Colman has accurately described the microscopical appearances of these little growths.[3] There is a cone of ordinary unstriped muscle covered by a thick layer of mucous membrane which consists of Lieberkühn's follicles and adenoid tissue, exactly like the mucous membrane of the small intestine (cf. Case I). Various ingenious pathological theories have been advanced to account for this development and extrusion of the omphalo-mesenteric duct at the umbilicus, while it disappears wholly or partially elsewhere. There is, of course, something more than a persistence, and we are left to choose

[1] *Ibid.*, 1880, i. 701. [2] *Tumeurs de l'Ombilic*, 1886, p. 41 *et seq.*
[3] *Tr. Path. Soc. Lond.*, 1888, xxxix. 110.

between excessive development followed by involution of that portion of the tube which connects the polypus with the ileum, and development into a structure resembling small intestine at a particular point in the line of the duct. The fluid from these polypi closely resembles succus entericus, and its digestive properties should always be investigated.

Very rarely polypi have been described which microscopically have the structure of pyloric mucous membrane. (Tillmanns.[1] C. B. Ball.[2])

One cannot determine the exact share taken in producing these various abnormal conditions by remnants of the omphalomesenteric vessels. It is probable that they are most concerned in the production of bands or cords, especially those in or connected with the mesentery.

Finally, a process of super-involution of the vitelline duct has been supposed to account for cases of partial or complete occlusion of the small intestine, near the usual site of attachment of a Meckel's diverticulum.

### THE QUIET PRODUCTION OF ANÆSTHESIA.

[1] *Deutsche Ztschr. f. Chir.*, Bd. xviii. [2] *Illust. M. News*, 1889, iv. 149.

## Figures and Tables

**DIAGRAM 1. f1:**
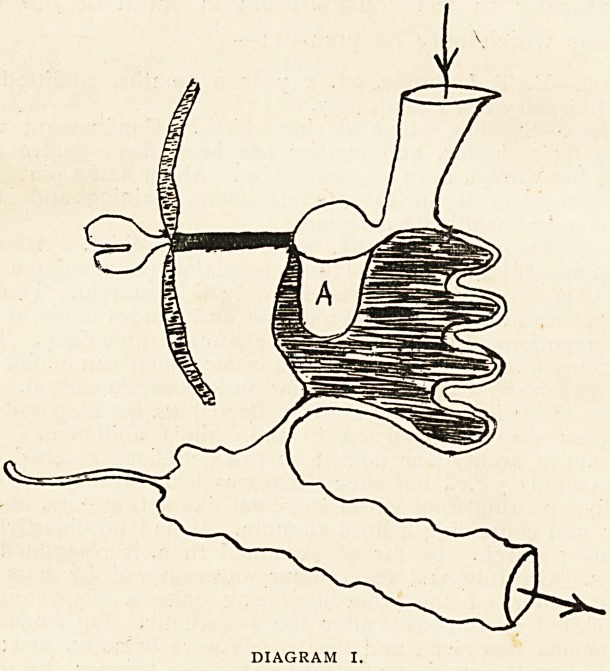


**DIAGRAM 2. f2:**
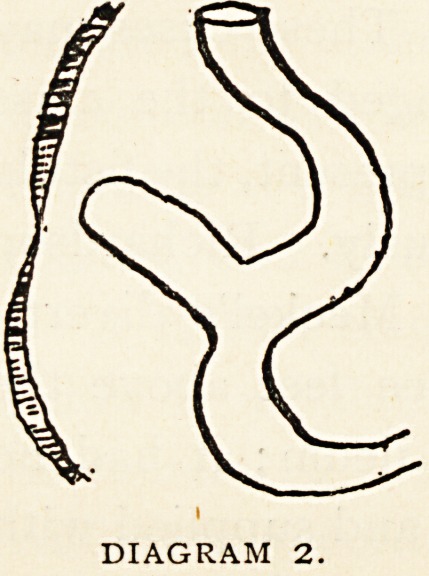


**DIAGRAM 3. f3:**
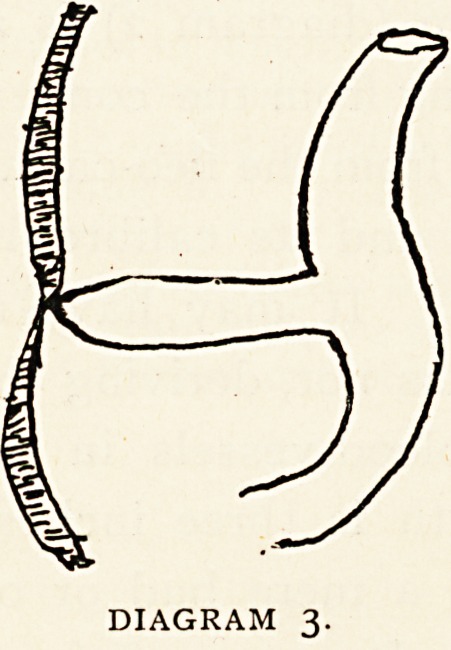


**DIAGRAM 4. f4:**
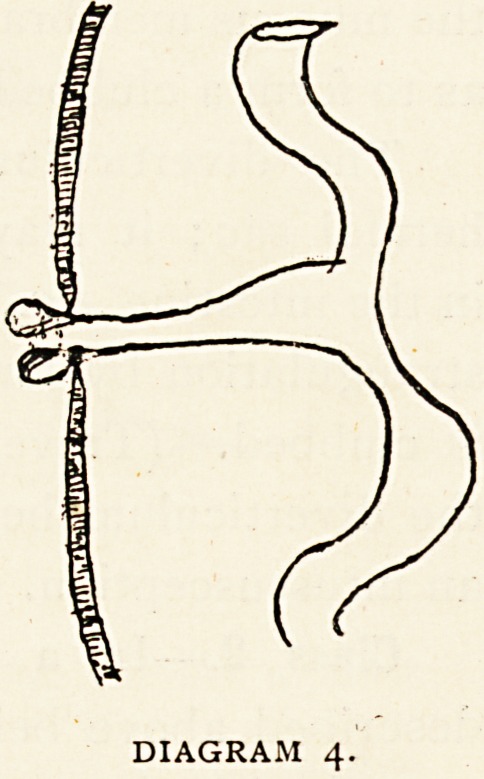


**DIAGRAM 5. f5:**
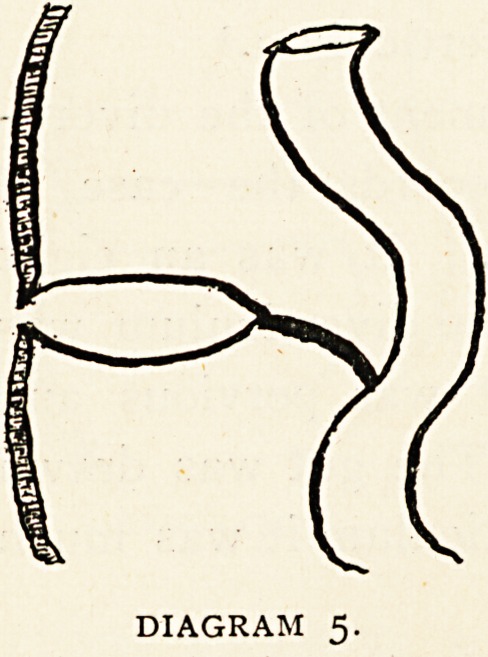


**DIAGRAM 6. f6:**
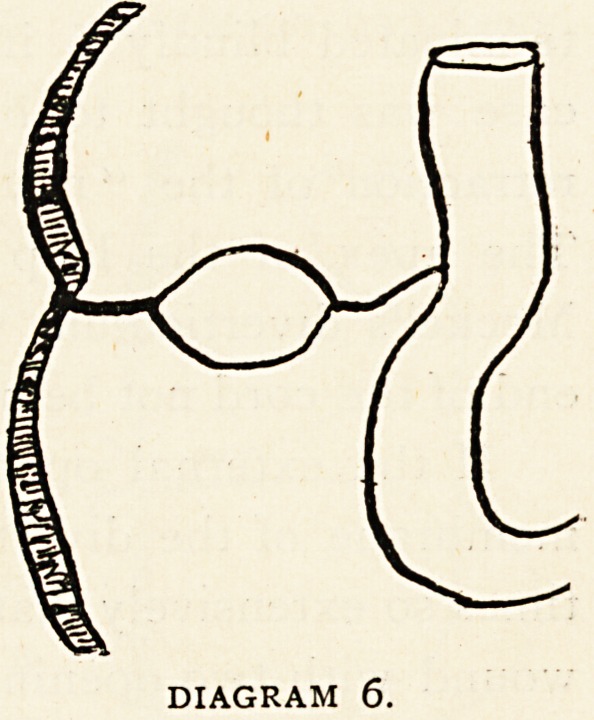


**DIAGRAM 7. f7:**
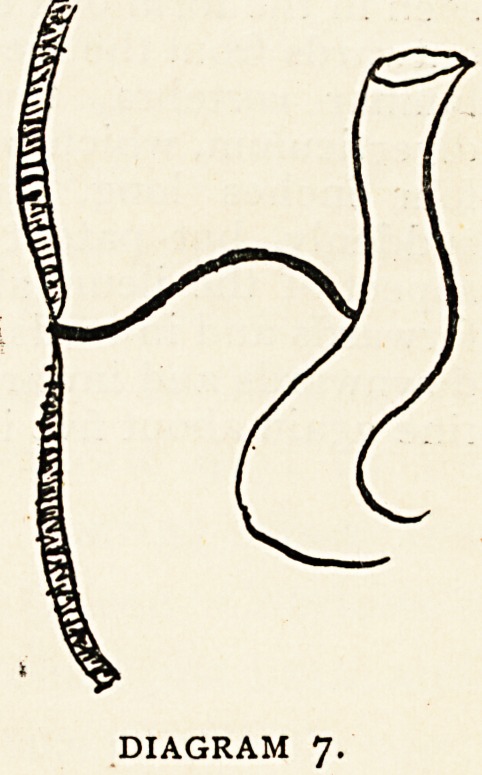


**DIAGRAM 8. f8:**